# Diagnostic Stability of Acute and Transient Psychotic Disorders at a Tertiary Care Center: A Retrospective Record-Based Study

**DOI:** 10.7759/cureus.77112

**Published:** 2025-01-07

**Authors:** Priya Kathfar, Priyash Jain, Deepti Rastogi, Vijay Niranjan

**Affiliations:** 1 Psychiatry, Mahatma Gandhi Memorial (MGM) Medical College, Indore, IND; 2 Pharmacology, Mahatma Gandhi Memorial (MGM) Medical College, Indore, IND

**Keywords:** acute and transient psychotic disorders, diagnostic stability, psychosis, schizophrenia, schizophrenia and other psychotic disorders

## Abstract

Background

Acute and transient psychotic disorders (ATPD) have been a diagnostic enigma due to their fleeting nature. While classified in various systems, discrepancies continue between the WHO's International Classification of Diseases, 10th Edition (ICD-10), International Classification of Diseases, 11th Edition (ICD-11), and Diagnostic and Statistical Manual of Mental Disorders, Fifth Edition (DSM-5). Research on ATPD, especially in developing countries like India, is scarce, leading to uncertainty about their prevalence and diagnostic stability.

Aim and objective

This study aims to investigate the stability of ATPD diagnoses over a period of one year in an Indian context.

Methods

A retrospective study conducted at a tertiary care center examined the diagnostic stability of ATPD. Fifty-four patients diagnosed with ATPD between January and June 2022 were identified from outpatient records. Their medical history, including age, sex, symptom onset, duration, stressors, and family history, was analyzed. Additionally, follow-up diagnoses at six months and one year were assessed to determine how often the initial ATPD diagnosis changed. Data analysis employed tools like Microsoft Excel (Microsoft Corporation, Redmond, Washington, United States) and IBM SPSS Statistics for Windows, Version 26.0 (Released 2019; IBM Corp., Armonk, New York, United States).

Results

Most patients diagnosed with ATPD were young women. Initially, at six months, the diagnostic stability was 59.25%, but this dropped to 40.74% after a year which was significant with a p-value of 0.031. Schizophrenia became the main alternative diagnosis, with bipolar disorder also increasing significantly over time.

Conclusions

While results showed higher initial stability than reported in developed countries, this stability significantly decreased within a year. Diagnostic shifts primarily led to schizophrenia or bipolar affective disorder. These findings suggest long-term follow-up is crucial for accurate prognosis and underscores the need for further research with larger samples and improved designs to refine diagnostic practices for ATPD.

## Introduction

Since the beginning of the 19th century, acute and transient psychotic disorders (ATPD) have posed a significant challenge in the context of their nosological position. Owing to their transient and unstable nature, this group of disorders has been difficult to study and, hence, has been repeatedly reconceptualized as "bouffée délirante", cycloid psychosis, reactive psychosis, and atypical psychosis. The conundrum of their classification continues today as the confusion has percolated into modern classification systems [1].

The WHO formulated the International Classification of Diseases, 10th Edition (ICD-10), proposing the diagnosis of ATPD under the broad category of "schizophrenia, schizotypal, and delusional disorders" with six subtypes. The diagnosis as per ICD-10 is described to be having acute or abrupt onset of psychotic symptoms with complete remission within 2-3 months of its onset [2]. However, in its recent 11th edition, the ICD has reduced the diagnosis to the ICD-10 equivalent of acute polymorphic psychotic disorder without the symptoms of schizophrenia which is characterized by hallucinations, delusions, and perceptual disturbances which are markedly variable, changing from day to day or even from hour to hour, and are accompanied with intense transient feelings of happiness and ecstasy or anxieties and irritability [3]. The Diagnostic and Statistical Manual of Mental Disorders, Fifth Edition (DSM-5), includes an equivalent diagnosis of brief psychotic disorders under the broad category of schizophrenia spectrum and other psychotic disorders [4]. The diagnosis of brief psychotic disorder is similar to the diagnosis of schizophrenia not including the negative symptoms and the duration of up to one month.

Due to the paucity of research in developing countries and the brevity of the disorder, the data on the incidence and prevalence of the disorder remains limited [5]. Community-based studies have not reported the prevalence of ATPD in the community samples. However, the prevalence of ATPD in outpatient settings has been reported to be 2.26%, while in emergency settings, it is reported to be 2.3% [6-8]. This lack of data on the prevalence of ATPD is also, in part, because of varying diagnostic stability. Studies have generally reported lower diagnostic stability in developed countries, while the diagnostic stability reported in developing countries is higher [9]. However, studies are lacking in the Indian subcontinent. This study was conducted to evaluate the diagnostic stability of ATPD in order to fill the dearth in the Indian context.

## Materials and methods

This study was conducted in the Department of Psychiatry of Mahatma Gandhi Memorial (MGM) Medical College, Indore, India, to evaluate the diagnostic stability of ATPD. A retrospective analysis was performed on medical records of new cases visiting the outpatient department (OPD) from January 2022 to June 2022. A purposive sampling method was used, and all the outpatient cases that met the inclusion records were selected.

New cases were defined as first-time consultations for any mental health concern. The inclusion criteria were new cases provisionally diagnosed with ATPD as per ICD-10 during the study period. Records with incomplete or missing information and patients with chronic unstable medical conditions (like uncontrolled diabetes mellitus or uncontrolled hypertension) were excluded. Out of 1,147 new patients screened, 67 met the inclusion criteria. Thirteen records were excluded due to incompleteness to prevent them from compromising with the study findings. This yielded a final sample size of 54 participants.

Data were extracted into a structured sociodemographic and clinical datasheet, including variables such as age, sex, illness characteristics (onset, duration, presence of stressors), family history of mental illness, and the initial diagnosis. Follow-up records were reviewed to assess diagnostic stability at six months and one year. Diagnosis at both time intervals was noted for each patient and whether the patient had been lost to follow-up. 

Initial evaluations were conducted by trainee psychiatrists and reviewed by senior psychiatrists. Follow-up diagnoses were recorded on subsequent evaluations by a senior psychiatrist based on ICD-10. Trainee psychiatrists are the doctors currently undergoing their postgraduate training in psychiatry, while senior psychiatrists are the doctors who have completed their psychiatry postgraduate training. The study was exempted from approval by the Institutional Ethics Committee as it is a record-based study.

Statistical analysis

Data management was performed using Microsoft Excel, Version 2311 (Microsoft Corporation, Redmond, Washington, United States), and statistical analysis was conducted using IBM SPSS Statistics for Windows, Version 26.0 (Released 2019; IBM Corp., Armonk, New York, United States). Statistical analysis included the application of descriptive statistics to summarize demographic and clinical characteristics. The McNemar test was applied to test for the stability of ATPD diagnosis at six months to one year follow-up intervals.

## Results

As depicted in Table 1, the study included 54 participants with a mean age of 35.56 years (SD=17.46). The sample was nearly balanced between men (n=25; 46.3%) and women (n=29; 53.7%). Acute onset was the most commonly reported onset of illness (n=40; 74.08%), with financial setbacks and family conflict being the most frequent stressors. A family history of mental illness was present in a minority of participants (n=10; 18.52%), with psychotic disorders being the most common reported type. 

**Table 1:** Sociodemographic and clinical parameters of the participants SD: standard deviation

Sociodemographic and clinical parameters	N (%)
Age distribution mean (SD)=35.56 years (17.46)	
<20	11 (20.37)
20-29	17 (31.48)
30-39	12 (22.22)
40-49	7 (12.96)
50-59	2 (3.7)
60-69	3 (5.56)
70+	2 (3.7)
Sex	
Male	25 (46.3)
Female	29 (53.7)
Onset	
Abrupt	14 (25.92)
Acute	40 (74.08)
Stressor	
Post-partum	1 (1.85)
Financial setback	3 (5.56)
Family conflict	2 (3.7)
Others	3 (5.56)
None	45 (83.34)
Family history	
Psychotic disorder	7 (12.96)
Mood disorder	3 (5.56)
None	44 (81.48)

Table 2 lists the revised diagnosis at the six-month follow-up period. At the six-month follow-up, more than half of the cases (n=32; 59.25%) maintained the same diagnosis of ATPD. Schizophrenia was the diagnosis most cases converted to (n=12; 22.23%), followed by bipolar affective disorder (BPAD) (n=3; 5.56%). Three, i.e., 5.56% of the participants, were lost to follow-up.

**Table 2:** Diagnostic revision at the six-month follow-up

Six-month follow-up	Frequency	Percent
Stable	32	59.25
Schizophrenia	12	22.23
Bipolar affective disorder	3	5.56
Depression	2	3.7
Relapsed	2	3.7
Loss to follow-up	3	5.56
Total	54	100

Table 3 lists the revised diagnosis at a one-year follow-up interval which depicts that the majority of the cases were found to have a stable diagnosis of ATPD (n=22; 40.74%). Schizophrenia and BPAD were the first and second most common revised diagnoses (n=12 (22.23%) and n=8 (14.81%), respectively). The rate of conversion of diagnosis to BPAD doubled at the one-year period compared to the six-month follow-up. Nine patients, i.e., 16.67%, were lost to follow-up.

**Table 3:** Diagnostic revision at the one-year follow-up

One-year follow-up	Frequency	Percent
Stable	22	40.74
Schizophrenia	12	22.23
Bipolar affective disorder	8	14.81
Depression	2	3.7
Relapsed	1	1.85
Loss to follow-up	9	16.67
Total	54	100

As depicted in Table 4, a significant proportion of patients (six out of 34) no longer met the criteria for ATPD at one year despite being diagnosed at six months. The results were statistically significant with a p-value of 0.031. This finding supports the transient nature of ATPD in many cases, as more patients recovered by the one-year mark.

**Table 4:** The McNemar test of significance of patients diagnosed with ATPD at the six-month and one-year follow-up p-value=0.031 ATPD: acute and transient psychotic disorders

ATPD at the six-month follow-up	ATPD at the one-year follow-up	
	Yes	No
Yes	14	6
No	0	14

## Discussion

The current retrospective study was conducted in a tertiary care institute with the aim of evaluating the diagnostic stability of patients diagnosed with ATPD. ATPD as a diagnosis has been questioned for a long duration owing to their transient nature and lesser diagnostic stability. In agreement with the established literature, the prevalence of ATPD was slightly higher in women at 53.7% in comparison to men [10-12]. A similar distribution was noted in a study by Aadamsoo et al. which reported 60% of patients to be females and Rusaka and Rancāns which reported 60.7% to be females [13,14]. The average age at first psychotic episode in our study was higher in women than in men which is again in concordance with other studies such as Rusaka and Rancāns which reported an average age for females as 40.2 years while for males 29 years [14].

Various studies in developed countries have reported low diagnostic stability of ATPD. In a study by Aadamsoo et al., diagnostic stability was reported to be 34% by the end of the two-year follow-up period [13]. In another study, diagnostic stability was reported to be 39% by the end of the five-year follow-up period [12]. While developed countries have reported lower diagnostic stability, developing countries like India, Iran, and Egypt have reported higher diagnostic stability [9,11,15]. In this study, the diagnostic stability was high at 59.25% at the six-month follow up which then dropped down to 40.74% at the one-year follow-up.

Wherever the diagnosis was not stable, the shift was towards schizophrenia (22.23%) followed by BPAD (14.81%). This diagnostic change is similar to a study by Castagnini et al. which reported a shift towards schizophrenia to be 32.3% while towards bipolar it was reported to be 5.5% [12]. The findings are consistent with other studies like Mukherjee et al. where the majority of cases of ATPD shifted to schizophrenia (22%) followed by bipolar mood disorder (12.3%) [16]. The shift of ATPD to schizophrenia was even more pronounced in a study by Rusaka and Rancāns where this shift was reported to be at 70.7% [14]. These differences highlight the variability in diagnostic trajectories, possibly influenced by cultural, clinical, and systemic factors.

The progression from ATPD to schizophrenia or BPAD has significant implications for clinical practice. Early identification of patients at risk of transitioning to more chronic psychotic or mood disorders could enable targeted interventions. For instance, incorporating structured diagnostic tools or biomarkers in clinical settings might enhance diagnostic precision and allow for tailored treatment plans. Moreover, the diagnostic overlap between ATPD, schizophrenia, and bipolar disorder underscores the need for nuanced diagnostic frameworks. While ICD-10 offers a broader categorization of ATPD, ICD-11's more restrictive criteria may reduce misdiagnoses but risk excluding atypical presentations. Similarly, DSM-5's classification of brief psychotic disorder reflects different conceptualizations, complicating cross-system comparisons.

The limitation of the study was a small sample size of 54, which limits the statistical power of the study and increases the risk of random error. Also, the study was conducted in a single center which limits the generalizability of its results. The study also relies on retrospective design whereas a prospective study could have been more appropriate in the context. To address these concerns, future studies are required with better sample size and design.

The findings emphasize the importance of regular follow-ups for patients diagnosed with ATPD. Clinicians should remain vigilant about potential diagnostic shifts and consider the use of structured diagnostic tools to minimize variability. Training programs for mental health professionals should address the challenges of diagnosing transient psychotic disorders, particularly in resource-limited settings. Future studies should explore the biological and psychosocial factors underlying diagnostic transitions, including genetic predispositions, neuroimaging correlates, and the impact of environmental stressors. Comparative studies examining diagnostic stability across different healthcare systems could provide further insights into global disparities.

## Conclusions

This study investigated the diagnostic stability of ATPD in a tertiary care setting. While results showed higher initial stability than reported in developed countries, this stability significantly decreased within a year. Diagnostic shifts primarily led to schizophrenia or BPAD. These findings suggest long-term follow-up is crucial for accurate prognosis and underscores the need for further research with larger samples and improved designs to refine diagnostic practices for ATPD.
